# Two case reports and a systematic review of the literature on adult cerebral cortical encephalitis with anti-myelin oligodendrocyte glycoprotein antibody

**DOI:** 10.3389/fimmu.2023.1203615

**Published:** 2023-07-14

**Authors:** Meihui Xu, Chi Ma, Ming Dong, Chunjie Guo, Simin Yang, Yue Liu, Xu Wang

**Affiliations:** ^1^ Department of Neurology and Neuroscience Center, the First Hospital of Jilin University, Changchun, China; ^2^ Department of Neurosurgery, the First Hospital of Jilin University, Changchun, China; ^3^ Department of Radiology, the First Hospital of Jilin University, Changchun, China

**Keywords:** adult, cerebral cortical encephalitis, myelin oligodendrocyte glycoprotein antibody, myelin oligodendrocyte glycoprotein antibody-associated disease, MRI

## Abstract

**Background and purpose:**

Myelin oligodendrocyte glycoprotein antibody-associated disease (MOGAD) has gained recognition in recent years as an immune-mediated inflammatory demyelinating disease of the central nervous system. The clinical features and prognosis of MOGAD adult cerebral cortical encephalitis (adult CCE) have not been fully elucidated. This study aims to further characterize the clinical symptoms, magnetic resonance imaging (MRI) findings, and prognosis of CCE with anti-MOG antibody.

**Methods:**

We present two adult cases of CCE with anti-MOG antibody and summarize the clinical symptoms, magnetic resonance imaging (MRI) findings, and prognosis of this phenotype as per a completed systematic review of the literature.

**Results:**

We found a total of 39 cases of MOGAD adult CCE (36% females; average age of onset of 29 years). Among them, 85% had seizure, 82% had headache, 64% had cortical symptoms, 64% had fever, 54% had changes of consciousness, and 38% had ocular symptoms. All cases demonstrated cerebral cortical T2 fluid-attenuated inversion recovery (FLAIR) lesions on MRI. Of the 25 patients (with seizure or not) who had EEG reports, 76% of patients showed abnormal EEG. Cerebrospinal fluid (CSF) white blood cell count of 90% of patients and CSF total protein of 67% of patients were elevated. In 16 patients with available CSF cytology data, 11 (69%) had abnormal cytology findings with monocytic predominance. In the 15 cases for which MOG antibody IgG was tested in both serum and CSF, 14 (93%) demonstrated a higher positive MOG IgG titer in serum than CSF. The majority of patients were treated with immunosuppressive therapy (97% corticosteroids, 15% mycophenolate mofetil, 13% IVIg, 5% azathioprine, and 5% other). The majority of patients had a favorable prognosis after treatment, as exemplified by improved clinical symptoms and imaging. Two patients relapsed.

**Conclusions:**

The clinical presentation and prognosis of adult CCE remain less understood in comparison to more common MOGAD phenotypes. It is important to consider MOGAD as an underlying etiology for adult CCE, as early detection and immunotherapy may improve outcomes.

## Introduction

MOGAD is a recognized immune-mediated central nervous system inflammatory demyelinating disease. It is an independent spectrum of disease distinct from multiple sclerosis (MS) and neuromyelitis optica spectrum disorder (NMOSD). In 2017, Ogawa et al. (2017) proposed a unique clinical type: benign unilateral cortical encephalitis, which presented with epileptic seizures in the context of positive MOG antibodies (1). Since this initial description, further case reports and cohort studies describing CCE in MOGAD have been published (2–6). A systematic review of the literature of published cases revealed that clinical manifestations of CCE with anti-MOG antibody are diverse. The recognized diversity of the clinical presentation of MOGAD adult CCE highlights a need to differentiate this process from alternative diagnoses such as MS, NMOSD, leukodystrophies, and others. Herein, we report two cases of adult CCE with anti-MOG antibody positivity in conjunction with a systematic review of pertinent literature.

## Case 1

**Figure 1:**
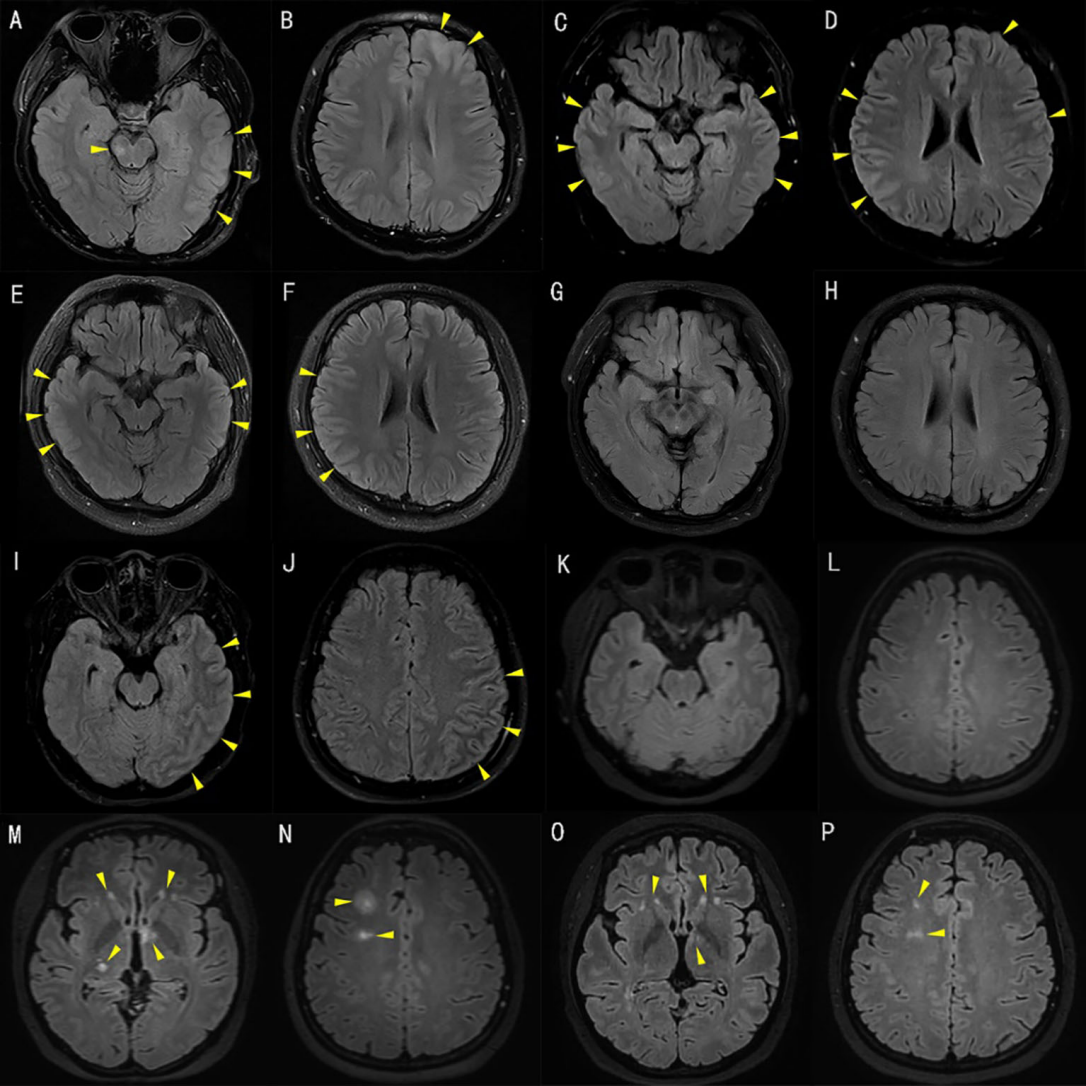
T2 fluid-attenuated inversion recovery (FLAIR) of brain magnetic resonance imaging (MRI). [A–H patient 1, I–P patient 2]. T2 FLAIR brain MRI of patient 1 at initial presentation demonstrated hyperintensities involving the brainstem, left frontal, parietal, and temporal cortex **(A, B)**. T2 FLAIR brain MRI after admission to our hospital and suffering from status epilepticus demonstrated hyperintensities involving the left frontal cortex and bilateral temporo-parietal cortex **(C, D)**. Repeat T2 FLAIR brain MRI three weeks after immunotherapy initiation showed improvement in the bilateral temporo-parietal cortex **(E, F)**. There were no obvious lesions on images seven months after the initial treatment **(G, H)**. Axial T2 FLAIR brain MRI of patient 2 at initial presentation demonstrated hyperintensities involving the left temporal, parietal, and occipital cortex **(I, J)**. T2 FLAIR brain MRI ten days after immunotherapy initiation was normal **(K, L)**. T2 FLAIR brain MRI after disease relapse demonstrated multiple hyperintensities, in keeping with acute disseminated encephalomyelitis involving the bilateral thalamus, basal ganglia, and right centrum semiovale **(M, N)**. At re-assessment four months after second discharge, the patient was clinically asymptomatic with improving FLAIR lesions on repeat T2 FLAIR brain MRI **(O, P)**.

A 24-year-old man (no past neurologic history) presented with persistent neck stiffness and occipital pain without known cause. Approximately one week later, he was admitted to a local hospital for management of status epilepticus. He required endotracheal intubation and there was no significant improvement in seizure control despite pharmacologic sedation. CSF examination showed pleocysis (310×10^6^/L, normal range 0~8×10^6^/L), normal protein (38 mg/dl), and elevated glucose (5.0 mmol/L). The autoimmune encephalitis antibodies (including anti N-methyl-D-aspartate receptor antibody, anti-contactin-associated-protein-like 2 antibody, anti-leucine-rich gliomain activated 1 antibody, anti-a-amino-3-hydroxy-5-methyl-isoxazo-lepropionic acid receptor antibody, anti-γ-aminobutyric acid-B receptor antibody, anti-IgLON5 antibody, anti-D2R antibody, and anti-DPPX antibody) in serum and CSF and virus antibodies (including HSV-I-IgG, HSV-I-IgM, HSV-II-IgG, HSV-II-IgM, TOX-IgG, TOX-IgM, CMVIgG, CMV-IgM, RVIgG, RVIgM, EBV capsid antigen antibody IgG, IgM and IgA, EBV early antigen antibody IgG, IgM, and IgA) in CSF were all negative. Serum leukocytosis was present (white blood cell count 22×10^9^/L, normal range 3.5~9.5×10^9^/L). T2 FLAIR brain MRI revealed hyperintense lesions involving the brainstem, left frontal, parietal, and temporal cortex, see **Figures 1A, B**; there was no contrast enhancement. A second lumbar puncture was then performed in order to observe changes in CSF, revealing persistent yet increasing leukocytosis (26×10^6^/L) and normal protein (32 mg/dl). Empiric treatment for viral encephalitis was initiated with penciclovir, sodium valproate, dexamethasone, dehydrant, and symptomatic supportive treatment. After discharge, clinically significant diaphoresis and subjective concerns regarding fever remained, prompting re-admission; he was then transferred to our hospital with a temperature of 38.4°C. A repeat CSF examination revealed lymphocytic pleocytosis (293×10^6^/L, 77% lymph) and elevated protein (76 mg/dL). The following day, recurrent status epilepticus ensued. Brain MRI showed FLAIR hyperintense lesions in the left frontal cortex and bilateral temporo-parietal cortex, see **Figures 1C, D**. A fourth CSF examination three days later revealed leukocytosis (436×10^6^/L) and elevated protein (116 mg/dl), and cytological classification showed 35% lymphocytes, 55% neutrophils, and 10% monocytes. Central nervous system demyelinating autoantibodies (including anti-AQP4, anti-MOG, and anti-MBP) testing was performed. Serum and CSF anti-MOG antibodies were positive at titers of 1:100 and 1:10 (CBA), respectively. Anti-AQP4 was negative in the serum and CSF. The autoimmune encephalitis antibodies (including anti-N-methyl-D-aspartate receptor antibody, anti-contactin-associated-protein-like 2 antibody, anti-Leucine-rich glioma inactivated 1 antibody, anti-a-amino-3-hydroxy-5-methyl-isoxazo-lepropionic acid receptor antibody, and anti-_γ_-aminobutyric acid-B receptor antibody), paraneoplastic syndrome antibodies (including anti-Hu, anti-Yo, anti-Ri, anti-CV2_(CRMP5)_, anti-Amphiphysin, anti-Ma1, anti-Ma2, anti-SOX1, anti-Tr_(DNER)_, anti-Zic4, anti-GAD65, anti-PKCγ, anti-Recoverin, and anti-Titin_(MGT30)_), and anti-ganglioside antibodies of serum (including anti-Sulfatide, anti-GM1, anti-GM2, anti-GM3, anti-GM4, anti-GD1a, anti-GD1b, anti-GD2, anti-GD3, anti- GT1a, anti-GT1b, and anti- GQ1b) were all negative. A diagnosis of MOGAD was concluded. Treatment was initiated with intravenous immunoglobulin 0.4 g/kg for 5 days and IV methylprednisolone (IVMP) 1 g/d for 3 days and the dose halved every 3 days. Oral prednisone tablets (1 mg/kg/d) were administered following the IVMP course. Two weeks later, repeat testing showed a decrease in MOG serum and CSF titers to 1:10 and 1:1, respectively. Electroencephalogram (EEG) demonstrated interictal, irregular sharp waves in the left temporal and right frontal regions. Repeat brain MRI three weeks from immunotherapy initiation showed improvement in FLAIR hyperintensity, see **Figures 1E, F**; there was no contrast enhancement. The visual evoked potential (VEP) and MoCA scale were normal. He was discharged with oral prednisone tablets (1 mg/kg/d, reduced by 1 tablet every two weeks), with ongoing clinical improvement. There were no obvious lesions on images seven months after the initial treatment, see **Figures 1G, H**, and the MOG antibody titer decreased (serum and CSF at titers of 1:1 and 1:1, respectively). After completion of a course of steroid tablets, he remains clinically improved without relapse and MOG antibody negative eleven months after the start of treatment.

## Case 2

A 31-year-old woman (no past neurologic history) suffered from sudden headache without obvious cause. She was admitted to the local hospital with fever, nausea, and vomiting a week later, and empiric antibiotics were prescribed for concern regarding a bacterial infection. Three days later, she returned due to worsened headache. CSF analysis revealed elevated opening pressure (225 mmH_2_O), leukocytosis (465x10^6^/L, normal range 0~8×10^6^/L), elevated protein (92 mg/dL), and normal glucose. Serum leukocytosis was also present (16x10^9^/L, normal range 3.5~9.5×10^9^/L). Empiric antivirals were initiated for presumed viral meningitis. Rapid clinical deterioration to unconsciousness ensued over a few hours. Repeat lumbar puncture revealed worsened opening pressure elevation (400mmH_2_0), persistent pleocytosis (208 cells/uL), and persistent elevated protein (72 mg/dL). On the third day of admission, she was aware after supportive treatment and physical examination showed positive Kernig sign. This patient did not have papilledema. Brain MRI showed FLAIR hyperintensity in the left temporal, parietal, and occipital cortex and there was no contrast enhancement, see **Figures 1I, J**. The MOG antibody titer in the serum was positive at a titer of 1:10 (CBA). She was diagnosed with unilateral cortical encephalitis with anti-MOG antibody. Empiric IVMP (1 g/d for 3 days, dose halved every 3 days) was initiated with subsequent clinical improvement. On the 13th day of admission, the repeated LP pressure was 243 mmH_2_O, and CSF examination revealed elevated leukocytes (24×10^6^/L) and protein (31 mg/dL). Repeated brain MRI showed no obvious lesions, see **Figures 1K, L**. She was discharged on oral prednisone tablets (1 mg/kg/d, reduced by 1 tablet every two weeks). The patient self-discontinued oral prednisone due to side effects and in this setting had disease relapse manifesting as paresthesias, blurry vision, and ataxia approximately 2 months later. Brain MRI showed multiple FLAIR hyperintensity, in keeping with acute disseminated encephalomyelitis involving the bilateral thalamus, basal ganglia, and right centrum semiovale, see **Figures 1M, N**. The MOG antibody titer in the serum was 1:10. IVMP again resulted in clinical improvement, and she was discharged with oral prednisone tablets (1 mg/kg/d, reduced by 1 tablet every two weeks) and mycophenolate mofetil (three tables each time, taken two times daily). At re-assessment four months after discharge, she was clinically asymptomatic with improving FLAIR lesions on repeat brain MRI, see **Figures 1O, P**. The prednisone course was completed after three months of therapy, and she remains on mycophenolate mofetil monotherapy. A repeat serum MOG titer three months after completion of the steroid course was 1:32.

## Methods

We searched PubMed for ‘[encephalitis] AND [MOG]’ and ‘[cortical] AND [MOG]’ to review the literature for cases of adult cortical encephalitis with anti-MOG antibody. All relevant published articles from 2017 through 2022 were reviewed for potential study inclusion. Cases were included if they (a) had predominantly cortical T2-FLAIR hyperintense lesions, (b) MOG-IgG antibodies were positive by CBA in serum and/or CSF, (c) were older than 18 years, and (d) excluded other infectious/autoimmune cases. Cases were excluded if available data were not sufficiently reported in the publication. Discrepancies between authors regarding the inclusion of cases were resolved by discussion. Information on the selected cases and our cases are presented in **Table 1**.

**Table 1:** Summary of the characteristics of 39 cases of MOGAD adult cerebral cortical encephalitis.

Case (Reference)	Age/Sex	Clinical Manifestations						Imaging	EEG	CSF WBC cell (mono:poly)	Protein (mg/dL) (CSF)	MOG (serum)	MOG (CSF)	Treatment	Relapse
		Headache	Fever	Seizure	ND	CD	VS								
**1 (** 1)	M/38	—	—	+	+	+	+	Unilateral	Slow waves	29(80:20)	35	1:512	1:32	IVMP, oral prednisone	No
**2 (** 1)	M/36	—	—	+	+	+	+	Unilateral	Normal	63(98:2)	38	1:2048	1:4	IVMP, oral prednisone	No
**3 (** 1)	M/23	+	—	+	—	+	—	Unilateral	Slow waves	101(51:49)	86	1:256	1:16	DEX, oral prednisone	No
**4 (** 1)	M/38	+	—	+	+	+	—	Unilateral	—	311(41:59)	53	1:1024	—	IVMP	No
**5 (** 3 **)**	M/23	+	+	+	+	—	—	Unilateral	Slow waves	85	Elevated	Positive	Positive	IVMP, oral prednisone	No
**6 (** 7)	M/24	+	+	+	+	+	—	Bilateral	Focal discharge	436	116	1:100	1:10	IVIg, IVMP, oral prednisone	No
**7 (** 7)	M/25	+	+	+	+	—	—	Unilateral	—	219	68	1:100	1:10	IVIg, IVMP, oral prednisone	No
**8 (** 8 **)**	F/29	—	+	+	+	+	+	Bilateral	Delta waves	73(mono)	—	1:512	—	IVMP, oral prednisone	No
**9 (** 9 **)**	F/29	+	+	+	—	—	—	Unilateral	—	349	74	—	Positive	IVMP, oral prednisone	No
**10 (** 10 **)**	F/19	+	+	+	+	+	—	Unilateral	High-voltage slow waves	200(72:28)	56	1:256	1:128	IVMP, oral prednisone	No
**11 (** 11)	M/27	+	+	+	+	+	—	Bilateral	Left amplitude is lower	205(mono 67%)	84	1:1024	—	IVMP, oral prednisone	No
**12 (** 12)	F/39	+	+	—	+	+	+	Unilateral	—	64	49	—	1:8	IVMP, oral prednisone	No
**13 (** 13)	F/31	+	—	+	—	—	—	Unilateral	Epileptiform abnormalities	Normal	46	1:10	—	IVMP, oral prednisone, MM	No
**14 (** 14)	M/19	+	+	+	—	—	+	Unilateral	—	120	49	1:32	—	IVMP, oral prednisone, MM	No
**15 (** 14)	M/23	+	—	+	+	+	—	Unilateral	—	Elevated	77	1:32	1:1	IVMP, oral prednisone, MM	No
**16 (** 15)	F/31	+	+	+	—	+	—	Bilateral	Cerebral dysfunction	Normal	Normal	Positive	—	IVMP, oral prednisone, azathioprine	No
**17 (** 16)	F/18	—	—	+	+	+	+	Unilateral	Slow waves	132	—	1:2560	1:64	IVMP, oral prednisone, rituximab	No
**18 (** 17)	M/23	+	+	+	+	+	—	Unilateral	Delta activity	57(15:85)	36	1:100	—	IVMP, oral prednisone	No
**19 (** 18)	F/19	+	+	+	—	—	+	Unilateral	Normal	46(mono)	54	1:512	—	IVMP, oral prednisone	—
**20 (** 19)	F/36	+	—	+	+	—	—	Unilateral	Slow waves	25lymp (lymp)	—	Positive	—	IVMP, oral prednisone, adalimumab	No
**21 (** 20)	M/37	+	+	—	+	+	+	Unilateral	Slow waves	164 (34:66)	83.3	1:2048	1:64	IVMP, oral prednisone	No
**22 (** 21)	M/34	+	—	+	+	—	+	Unilateral	—	411 (lymp 62%)	Elevated	Positive	Positive	IVMP, oral prednisone	No
**23 (** 22)	M/20	+	+	+	+	+	—	Bilateral	Normal	80	48	1:10	—	IVMP, oral prednisone, MM	No
**24 (** 22)	M/20	—	—	+	—	—	—	Unilateral	Sharp-slow waves	31	Normal	1:100	—	IVMP, oral prednisone, MM	No
**25 (** 23)	M/30	+	+	+	+	+	—	Unilateral	Slow waves	324 (mono 83%)	108	Positive	—	IVMP, oral prednisone	No
**26 (** 24)	M/23	+	—	+	+	—	+	Unilateral	Epileptic wave	32 (lymp69%)	54	1:1000	—	IVMP, oral prednisone	No
**27 (** 25)	F/29	+	—	—	+	—	—	Unilateral	Normal	37 (27%poly)	61	Positive	—	IVMP	—
**28 (** 26)	M/32	+	+	+	—	—	—	Bilateral	—	46	Normal	1:10	Negative	IVMP, oral prednisone, MM	No
**29 (** 26)	M/28	+	+	+	—	+	+	Unilateral	—	105	Normal	1:10	—	IVMP	No
**30 (** 27)	F/22	+	+	+	—	—	+	Unilateral	Normal	92 (mono 47%)	55.7	1:1024	—	Supportive treatment	No
**31 (** 28)	M/31	+	—	—	—	—	—	Unilateral	Slow wave	114 (90:10)	48.5	1:1024	1:128	IVMP, oral prednisone	No
**32 (** 18)	F/19	+	+	+	—	—	+	Unilateral	Normal	46 (mono)	54	1:512	—	IVMP, oral prednisone	—
**33 (** 29)	M/44	—	+	+	+	—	—	Unilateral	—	43	58.2	Positive	—	IVMP, oral prednisone	No
**34 (** 29)	F/52	+	—	+	+	+	—	Unilateral	—	12	26.9	Positive	—	IVMP, oral prednisone, azathioprine	No
**35 (** 30)	M/39	+	+	+	+	—	+	Bilateral	—	—	—	Positive	—	IVIg, IVMP	No
**36 (** 31)	M/55	+	+	—	+	+	+	Unilateral	Slow, Epileptic waves	2	48	1:80	1:40	IVIg, IVMP, oral prednisone	Yes
**37 (** 32)	M/26	+	+	+	—	—	—	Unilateral	—	692	151	1:512	1:32	IVMP, oral prednisone	No
**38 (Case 1)**	M/24	—	+	+	+	+	—	Bilateral	Sharp or sharp slow waves	720 (polycyte)	38	1:100	1:10	IVIg, IVMP, oral prednisone	No
**39 (Case 2)**	F/31	+	+	—	—	+	—	Unilateral	—	465 (lymp 65%)	92	1:10	1:10	IVMP, oral prednisone	Yes

ND, neurological deficits; CD, consciousness or dysphrenia; VS, visual symptoms; MM, mycophenolate mofetil; “—”indicates negative in clinical manifestations and no clinical data in other items.

## Results

A systematic literature review yielded 37 total cases meeting the inclusion criteria for our study of MOGAD adult CCE, supplemented by the 2 unique cases from our institution. Among these 39 cases, there was a slight male predominance (25M:14F), with an average age at clinical presentation of 29 years. Prominent clinical features were seizure (85%), headache (82%), focal neurologic deficits (64%), fever (64%), altered mentation (54%), and ocular symptoms ranging from eye pain or photophobia to vision changes (38%). All cases demonstrated abnormal radiographic findings on brain MRI. Among these, T2 FLAIR hyperintense lesions were more commonly unilateral (79%), although bilateral hemispheric lesions were present in others (21%), with less common brainstem or corpus callosal involvement. Of the 25 patients (with seizure or not) who had EEG reports, 76% of patients showed continuous EEG monitoring abnormal in the seizure phase, 12/20 showed slow waves, 6/20 showed epileptic waves, and 2/20 showed decreased amplitude and brain function. The CSF white blood cell count of 90% of patients and CSF total protein of 67% of patients were elevated. In 16 patients with available cytological data, 11/16 showed elevated CSF cytology with monocytes as the dominant cell and 5/16 had polykaryocytes as the dominant cell. In 15/39 patients whose data were available for both serum and CSF MOG antibody IgG, 14/15 serum antibody titers were higher than those of CSF. A total of 38/39 patients received IVIg, IVMP, or oral prednisone therapy, while one case only received supportive treatment, and 10/39 received immunosuppressants such as azathioprine, mycophenolate mofetil, rituximab, and adalimumab. Of the 39 reported cases, 2 cases (5%) relapsed, presenting with dizziness, memory impairment, slow responsiveness, paresthesia, blurry vision, ataxia optic neuritis, etc. Two patients relapsed and there was nothing unique about them. The relapse of case 2 was related to rapid discontinuation of oral prednisone, with a repeated MOG antibody titer in the serum of 1:10 (CBA), while, unfortunately, another patient who also relapsed after completion of appropriate oral prednisone tablets did not agree to undergo a MOG antibody titer assay again.

## Discussion

MOGAD is a CNS demyelinating disorder with a variable spectrum of clinical disease, in part understood by the various anatomical regions where myelin oligodendrocyte glycoprotein is present within the nervous system. It is exclusively expressed on the outer surface of the myelin sheath and plasma membrane of oligodendrocytes in the CNS. MOG can participate in microtubule stability as a regulator, can regulate immune responses as an activator of complement, and may have a significant role in cellular adhesion (33). The experimental autoimmune encephalomyelitis (EAE) model by Spadaro et al. (2018) (34) showed that the pathogenic mechanisms of MOG antibody were complicated. On the one hand, it can induce blood-brain barrier damage and the activation of macrophages as a result of its synergy with MBP-specific T cells and antibodies, eventually leading to demyelinating changes *via* cytotoxicity and complement activation. On the other hand, it can interact with MOG-specific T cells to enhance the infiltration and activation of T cells, thereby enhancing the inflammatory response. A brain biopsy performed before glucocorticord therapy in the case of Ikeda et al. (8) showed mild inflammatory changes in the cortex and sub-cortex, without distinct demyelination. In the case of Patterson et al. (12), a brain biopsy of the left parietal lobe and dura showed interstitial and perivascular lymphocytic infiltrates, with marked involvement of the vessel wall. These two brain biopsy cases did not present any evidence of demyelination. These pathological characteristics differed from the EAE model and those in previous reports on NMOSD, ADEM, and atypical MS. As mentioned by van der Valk et al. (35), in the process of myelin breakdown in MS, MOG is rapidly degraded, whereas the process for myelin basic protein (MBP) may take more time. The case reported by Ikeda et al. (8), demonstrating limited loss of MOG immunoreactivity, might suggest ‘pre-active’ lesions of demyelination. Fujimori’s case did not exhibit the loss of MOG, probably because biopsy was performed after steroid therapy, and the treatment may modify the pathology. Further studies are still needed to elucidate the pathogenesis of this phenotype.

The clinical manifestations of cerebral cortical encephalitis with anti-MOG antibody are not typical. Many cases were delayed or even misdiagnosed due to various reasons, such as the neglect of clinical symptoms, self-medication, lack of doctors’ theoretical knowledge, and so on. Our cases had clinical characteristics similar to infectious encephalitis; therefore, they were easily misdiagnosed initially without detection of anti-MOG antibody. A total of 9/39 patients were initially diagnosed with infectious encephalitis and were given antiviral or antibiotic treatment; however, the disease progressed during the treatment. In addition, a case (12) was misdiagnosed as central nervous system vasculitis, whose routine blood and cerebral imaging examination were normal, with cerebral biopsy showing lymphocyte infiltration in the small blood vessels and no obvious demyelinating lesions. The CSF MOG antibody was positive after retrospective analysis and the biopsy specimen showed no fibrinoid necrosis; the clinical diagnosis was then corrected. Therefore, it is of great significance to improve the detection of various examinations and tests, including head MRI and antibody, as soon as possible for early diagnosis of this disease. The rapid deterioration of our case 1 and the case by Hang Shu et al. (7) emphasizes the importance and necessity of early diagnosis and treatment.

In terms of imaging manifestations, it is necessary to distinguish whether MRI abnormalities are brain damage caused by epilepsy or MOG. In general, cortical damage caused by epilepsy is characterized by hyperintensity on diffusion-weighted MR images (DWI). Transient focal hyperintensity on DWI with corresponding reduction of the apparent diffusion coefficient (ADC) indicates cytotoxic edema, which is an increasingly recognized phenomenon in the phase of epileptic seizures (36), while CCE of MOGAD usually presents with cerebral cortical hyperintensity on FLAIR. Unilateral cerebral cortical lesions on FLAIR of MRI are more frequent, and cortical lesions may be accompanied by corresponding leptomeningeal enhancement (4). According to a literature review, CSF cytology of MOGAD-related CCE may be characterized by an inflammatory response dominated by neutrophils (37). Cerebrospinal fluid inflammation is easily confused with infectious diseases of the nervous system, and early diagnosis is difficult. Adequate etiological examination is needed to exclude infection. Since MOG-IgG is produced in the peripheral blood, serum is the preferred test sample, and CSF testing only provides supplementary information (37). The serum MOG-IgG titer was correlated with disease activity, and the titer was higher in the acute stage than in the remission stage. In addition, serum MOG-IgG titers were associated with treatment status and decreased after immunosuppression or plasmapheresis (38). In 15 available datasets for both serum and CSF MOG antibody IgG, the serum antibody titer was higher than that of CSF. Some studies have reported that the higher the antibody titer at the time of onset and the longer the duration of antibody positivity, the greater the likelihood of relapse (7). In addition, as a relapse biomarker, the expression of TNFAIP3 was downregulated during relapse as compared to remission in MOGAD patients (39).

The treatment response varies in cerebral cortical encephalitis with anti-MOG antibody. The first-line treatment of patients includes HIMP and IVIg in the acute stage, and significant improvements in clinical symptoms and imaging can usually be observed after steroid treatment. However, some patients relapse after withdrawal therapy, and therefore, patients may be treated with prolonged steroid tablets or immunosuppressive therapy, such as mycophenolate mofetil (MM), rituximad (RTX), and cyclophosphamide, to prevent relapse.

At present, a recommendation of diagnostic criteria for MOG encephalomyelitis (MOG-EM) has been proposed (38); however, there are no unified diagnostic criteria for CCE with anti-MOG antibody. There were a few cases of adult MOGAD with CCE reported internationally. We conducted a systematic review of the literature for a better understanding of this unique clinical phenotype. In general, clinical diagnosis must be made based on symptoms, signs, and auxiliary examinations. In particular, a diagnosis of adult CCE with anti-MOG antibody should be considered when young patients present with fever, headache, epilepsy, or unilateral (or bilateral) cortical or sulcus FLAIR hyperintensity. Timely detection of MOG antibody in serum is helpful for accurate diagnosis of this phenotype, avoiding misdiagnosis and unnecessary treatment.

## Data availability statement

The original contributions presented in the study are included in the article/supplementary material. Further inquiries can be directed to the corresponding author.

## Ethics statement

Written informed consent was obtained from the individual(s) for the publication of any potentially identifiable images or data included in this article.

## Author contributions

M-hX and YL collected the clinical data and reviewed the literature. M-hX and CM wrote the manuscript. C-jG and S-mY improve the quality of all the images. XW and MD critically revised the manuscript. All authors contributed to the article and approved the submitted version.
